# Effect of Nitric Acid-Modified Multi-Walled Carbon Nanotube Capping on Copper and Lead Release from Sediments

**DOI:** 10.3390/toxics13110912

**Published:** 2025-10-23

**Authors:** Xiang Chen, Dongdong Zhu, Xiaohui You, Yan Wang, Li Zhou, Xiaoshuai Hang

**Affiliations:** Nanjing Institute of Environmental Sciences, Ministry of Ecology and Environment, Nanjing 210042, China

**Keywords:** nitric-acid-modified multi-walled carbon nanotubes, sediments, copper, lead

## Abstract

Nitric acid-treated multi-walled carbon nanotubes (CNTs) have been extensively utilized for removing dissolved heavy metals from aqueous systems; however, their use as a capping material to immobilize heavy metals in sediments has rarely been investigated. Consequently, the impact of CNTs on millimeter-scale variations in pore-water heavy metal concentrations along sediment profiles remains poorly understood. In this study, CNTs were applied as a capping agent, and microelectrodes combined with high-resolution diffusive equilibrium in thin-film (HR-Peeper) samplers were employed to simultaneously obtain vertical profiles of pH, soluble copper (Cu) and lead (Pb), and dissolved oxygen (DO) in sediments in order to assess the effectiveness of CNTs in controlling the mobility of Cu and Pb. The results revealed that CNTs application markedly reduced the concentrations of soluble Cu and Pb, with maximum reduction rates of 58.69% and 64.97%, respectively. Compared with the control treatment, CNTs capping decreased the maximum release fluxes of soluble Cu and Pb by 3.78 and 1.91 µg·m^−2^·d^−1^, respectively. Moreover, CNTs treatment enhanced the stable fractions of Cu and Pb within sediments, thereby improving the sediment’s capacity to retain these metals. Overall, this study demonstrates that CNTs can serve as an effective capping material to inhibit the leaching of Cu and Pb from sediments, offering a promising strategy for the in situ remediation of heavy metal-contaminated sediments.

## 1. Introduction

The continuous expansion of global industrialization and urbanization has led to a persistent increase in heavy metal contamination in aquatic environments [1,2]. Heavy metals are toxic pollutants that are resistant to biological degradation [3,4]. They can bioaccumulate within organisms, posing serious risks to human health and threatening the stability of aquatic ecosystems [5]. Industrial effluents, urban runoff, and agricultural non-point sources are typically enriched with copper (Cu) and lead (Pb) [6,7]. After entering water bodies through outfalls or stormwater drains, these metals are rapidly adsorbed onto suspended particles and subsequently deposited in sediments, leading to elevated Cu and Pb concentrations in the sedimentary layer [6,7]. Particularly high concentrations of Cu and Pb have been reported near estuarine bay heads, urban embayment, and sewage outfalls [6,7]. Cu and Pb are among the most hazardous heavy metals, as they can impair the central nervous system and vital organs such as the lungs [8,9]. Although Cu is an essential trace element for humans, excessive or long-term exposure can cause hepatic, renal, hematological, and neurological disorders [10,11]. The World Health Organization (WHO) and the U.S. Environmental Protection Agency (EPA) have established maximum permissible Cu concentrations in drinking water at 2.0 and 1.3 mg L^−1^, respectively [12]. Pb, in contrast, is a systemic toxicant that accumulates in human tissues, especially bones [13,14,15]. It exhibits neurotoxicity, induces anemia and renal dysfunction, damages brain tissues, and can even be fatal at high exposure levels [13,14,15,16]. The WHO and EPA have set guideline values for Pb in drinking water at 0.01 and 0.015 mg·L^−1^, respectively [17]. Therefore, controlling Cu and Pb concentrations in aquatic systems is of great environmental and public health importance.

Sediments act as both sinks and secondary sources for heavy metals such as Cu and Pb in aquatic environments [18,19]. Variations in redox conditions and other physicochemical parameters at the sediment–water interface can trigger the remobilization and release of these metals, thereby threatening aquatic organisms [20]. Effective control of heavy metal release from sediments is therefore essential for maintaining water quality. Remediation of heavy metal-contaminated sediments generally relies on either in situ or ex situ approaches [5,21,22,23,24]. Although dredging can reduce metal concentrations in both water and sediments, it may also disturb sediment properties and promote the release of oxidizable metal species [5,25,26]. Consequently, in situ remediation technologies are increasingly favored for contaminated sediments [22,23,24]. While such approaches have shown success in immobilizing elements such as phosphorus, arsenic, tungsten, and cobalt, their effectiveness for Cu remains limited [22,23,24,27,28]. Hence, developing efficient capping materials for controlling Cu and Pb release from sediments is a pressing need.

Carbon nanotubes (CNTs) represent an emerging class of nanomaterials with promising environmental applications [29]. Based on their structure, they can be categorized into single-walled (SWCNTs) and multi-walled carbon nanotubes (MWCNTs) [29,30]. Owing to their large specific surface area, high porosity, and remarkable chemical stability, CNTs have found wide applications in biomedicine, mechanics, and electronics [29,30,31,32,33,34]. However, the atomically smooth surface of CNTs leads to strong intermolecular interactions, which cause the nanotubes to easily form poorly dispersed bundles or aggregates [29]. This reduces their contact area with contaminants, limiting their adsorption efficiency for heavy metals [29]. Furthermore, unmodified CNTs have a limited number of surface functional groups, which results in a low adsorption capacity for heavy metals [35,36]. To overcome this limitation, various modification techniques—both covalent and non-covalent have been developed [37]. Among them, acid oxidation is the most widely employed covalent method [29,38,39,40,41]. This treatment introduces surface functional groups such as hydroxyl, carboxyl, and carbonyl, thereby increasing surface area, enhancing wettability, and providing additional active sites for metal adsorption [38,39,40,41]. Numerous studies have demonstrated the potential of modified CNTs to remove heavy metals such as Cu and Pb from contaminated soils [42,43,44,45]. Nonetheless, research on the application of CNTs for sediment remediation remains limited [46,47,48,49]. Most existing studies have focused on mixing MWCNTs directly with sediments to assess their effects on cadmium (Cd) adsorption and on metal speciation transformations [46,47,48,49]. In contrast, investigations employing MWCNTs as capping materials—especially when encapsulated in permeable fabrics—to immobilize heavy metals in sediments are scarce. Therefore, further research is needed to explore the feasibility and efficiency of acid-oxidized CNTs in mitigating the release of Cu and Pb from sediments.

In this study, sediments from Meiliang Bay in Lake Taihu were used to investigate the performance of multi-walled carbon nanotubes (CNTs) as a capping material. Microelectrodes and high-resolution diffusive equilibrium in thin-film (HR-Peeper) samplers were employed to obtain in situ vertical profiles of pH, soluble Cu and Pb, and dissolved oxygen (DO) in the sediment. The specific objectives were to: (1) evaluate the feasibility of applying CNTs as a capping agent to suppress the release of Cu and Pb from sediments and quantify their removal efficiency, and (2) elucidate the mechanisms by which CNTs stabilize Cu and Pb within the sediment matrix. The findings are expected to provide a theoretical basis for developing in situ strategies to mitigate the leaching of Cu and Pb from lake sediments.

## 2. Materials and Methods

### 2.1. Study Area

**Figure 1:**
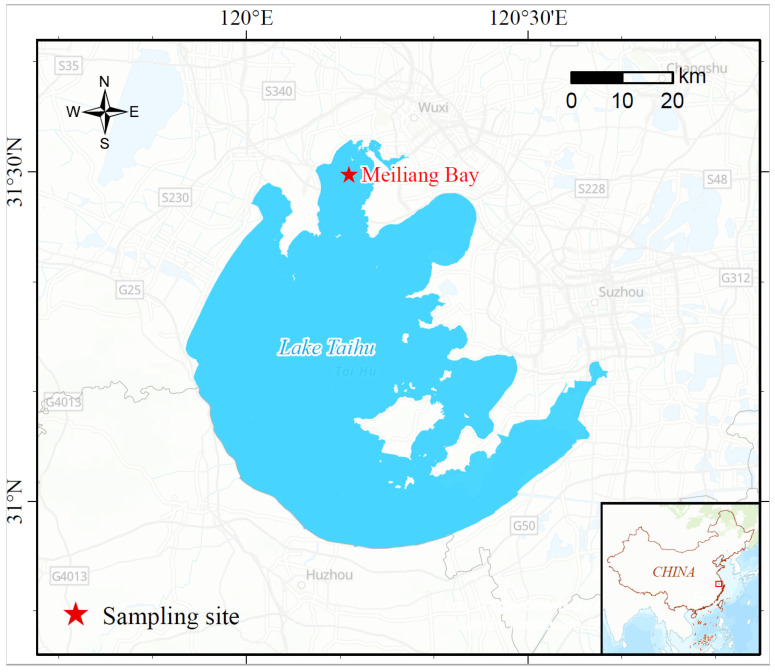
The sampling site in Meiliang Bay.

The northern section of Lake Taihu, known as Meiliang Bay (31°30′31.1″ N, 120°10′31.0″ E), covers approximately 123 km^2^ and has a water depth ranging from 1.8 to 2.3 m (Figure 1). Increasing anthropogenic activities around Lake Taihu have led to the discharge of untreated or insufficiently treated industrial, agricultural, and domestic wastewater into the lake, resulting in the enrichment of nutrients and heavy metals in the sediments [50,51,52].

### 2.2. Experimental Design

**Figure 2:**
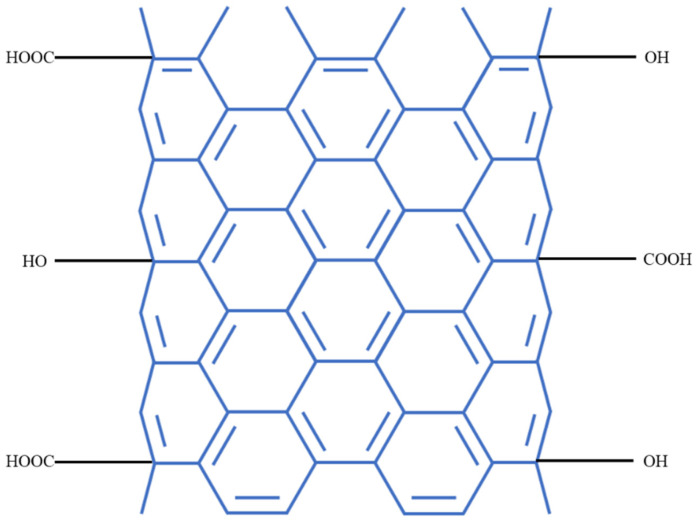
Schematic diagram of CNTs.

Six sediment cores were collected from Meiliang Bay using a gravity corer and immediately transported to the laboratory with the overlying water intact. Following the procedure described by Sun et al. [53], each core was sectioned at 2 cm intervals. Sediment slices from identical depths of all six cores were combined, homogenized, and passed through a 0.6 mm sieve to remove coarse debris and macro-benthos. The processed sediments were repacked into six acrylic columns (9 cm inner diameter, 30 cm height) to a depth of approximately 16 cm, maintaining the original layering structure. All columns were placed in a temperature-controlled water bath for 14 days of pre-incubation to restore sediment equilibrium. CNTs were obtained from Jiangsu XFNANO Materials Tech Co., Ltd. (Nanjing, China). The CNTs were treated with concentrated nitric acid (65%, Merck, Rahway, NJ, USA) for 20 h to introduce oxygen-containing functional groups. After treatment, the CNTs were repeatedly rinsed with deionized water until the rinse water reached a neutral pH, followed by oven drying at 110 °C for 24 h. The dried CNTs (Figure 2) were subsequently used as a capping material to remove heavy metal ions from sediments [54]. For the capping experiment, 5.5 g of CNTs were wrapped in permeable gauze and carefully placed on the sediment surface of three cores to form the CNTs-capped group, while the remaining three cores without CNTs coverage served as the control group.

### 2.3. Sample Collection

At 15, 45, and 90 days after CNTs capping, vertical profiles of dissolved oxygen (DO) and pH were measured in all sediment cores using microelectrodes. Following these measurements, high-resolution diffusive equilibrium in thin-film (HR-Peeper) samplers were inserted into the sediments and allowed to equilibrate for 48 h. Upon retrieval, pore-water samples were extracted, acidified with 3% HNO_3_, and analyzed for dissolved Cu and Pb using inductively coupled plasma–mass spectrometry (ICP–MS; NEXION 350X, PerkinElmer, Shelton, CT, USA). On day 90, the sediment cores were sectioned into layers of 0–1 cm, 1–2 cm, and 2–5 cm, freeze-dried, and subjected to the BCR sequential extraction procedure to determine the speciation of Cu and Pb [55]. The sequential extraction process for sediment Cu and Pb included the following steps: F1 (Weak-acid extractable Cu and Pb, 40 mL of 0.01 M CH_3_COOH, 25 °C, 16 h), F2 (Reducible Cu and Pb, 40 mL of 0.1 M NH_2_OH·HCl, pH = 1.5, 25 °C, 16 h), F3 (Oxidizable Cu and Pb, 5 mL of 8.8 M H_2_O_2_, pH = 2, 2 h at 85 ± 2 °C (near dryness), followed by 25 mL of 1 M NH_4_CH_3_COO, pH = 2, 25 °C, 16 h), and F4 (Residual Cu and Pb, 4.5 mL of HCl + 1.5 mL of HNO_3_, 120 °C, 1 h, with Residual Cu and Pb calculated as total Cu and Pb-F1-F2-F3).

### 2.4. Adsorption Experiments

Batch adsorption experiments were conducted to evaluate the adsorption performance of CNTs for Cu and Pb. Standard Cu and Pb solutions were prepared using analytical-grade CuCl_2_·2H_2_O and PbCl_2_ (Sinopharm Chemical Reagent Co., Ltd., Shanghai, China) at concentrations ranging from 10 to 500 mg·L^−1^. For each test, 40 mL of Cu or Pb solution and 0.1 g of CNTs were added to a 50 mL centrifuge tube. The mixtures were agitated at 25 °C for 24 h at 180 r·min^−1^ to reach adsorption equilibrium, followed by centrifugation at 3500 r·min^−1^ for 10 min. The supernatants were filtered through 0.45 μm syringe filters (Ronghua Co., Tianjin, China), and the residual Cu and Pb concentrations were determined by ICP–MS (NEXION 350X, PerkinElmer, Shelton, CT, USA).

### 2.5. Data Analysis

In this research, the fluxes of soluble Cu and Pb released at the interface between sediment and water in the columns were determined by applying Fick’s first law of diffusion, as expressed in the equation below:

$$F=\frac{-\varphi D_{0}}{1-ln\varphi^{2}}-\frac{\partial C}{\partial Z}$$

where φ denotes the sediment porosity; D_0_ represents the sediment diffusion coefficient of Cu and Pb; C denotes the soluble Cu and Pb concentrations (μg L^−1^); z denotes the sediment depth (mm); $(\frac{\partial C}{\partial Z}{)}_{z=0}$ indicates the soluble Cu and Pb concentration gradients in the pore water at the sediment-water interface (mm). All figures were generated using Origin 2017 software.

## 3. Results and Discussion

### 3.1. Adsorption Isotherms

**Figure 3:**
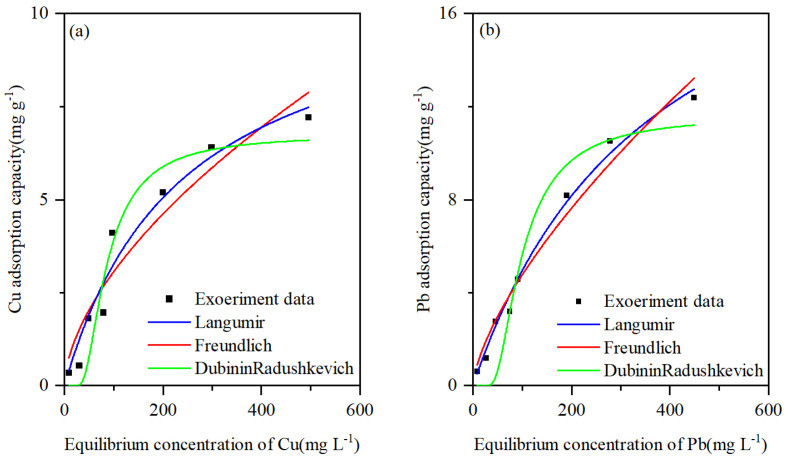
Adsorption isotherms of (**a**) Cu and (**b**) Pb from water on CNTs.

The adsorption isotherms for Cu and Pb adsorption onto CNTs are shown in Figure 3a,b. The adsorption capacity of CNTs for Cu and Pb increased with increasing Cu and Pb concentrations. The isotherm data were further analyzed using the Langmuir [56], Freundlich [57], and Dubinin–Radushkviech (DR) [58] equations. The results reported in Table 1 showed that the Langmuir equation was more suitable for Cu and Pb (Figure 3a,b). The maximum adsorption capacities (qm) of CNTs for Cu and Pb, calculated using the Langmuir isotherm model, were 11.076 and 23.206 mg g^−1^, respectively. A high K_f_ value calculated using the Freundlich model indicates that CNTs have a high affinity for heavy metal ions. The K_f_ values for Cu and Pb calculated by the Freundlich model are 0.201 and 0.21, respectively. This suggests that CNTs have a higher affinity for Pb compared to Cu. Additionally, the n values calculated by the Freundlich model for Cu and Pb are 1.695 and 1.473, respectively, both greater than 1, indicating that the adsorption of Cu and Pb by CNTs is favorable [54]. The E values calculated from the DR model constant (K_dr_) for the adsorbed Cu and Pb on CNTs were 23.271 and 20.143, respectively. This finding suggests that the adsorption of Cu and Pb by CNTs is mainly controlled by a chemisorption mechanism (Table 1) [58,59,60,61]. Indeed, E values < 8 kJ mol^−1^, 8–16 kJ mol^−1^, and >16 kJ mol^−1^ indicate physical adsorption, ligand exchange, and chemical adsorption mechanisms, respectively [58,60,61,62].

### 3.2. Sediment DO and pH Changes

**Figure 4:**
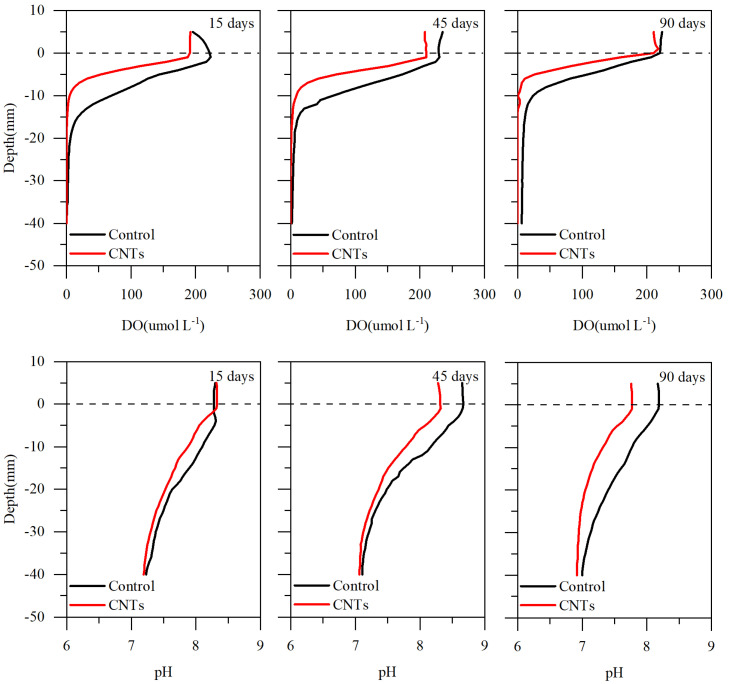
Vertical distribution of DO and pH in sediments of the control and CNTs groups at different sampling times.

Figure 4 depicts the fluctuations in DO and pH levels in the sediments. On days 15, 45, and 90 after CNTs application, DO levels in the CNTs group remained consistently lower than those in the control group. On days 15, 45, and 90, the DO concentrations in the overlying water of the control group were 209.33, 231.63, and 221.84 μmol L^−1^, respectively, whereas the corresponding values for the CNTs group were 191.42, 208.57, and 212.87 μmol L^−1^. Compared with the control, the CNTs capping reduced the DO concentration by 8.56%, 9.96%, and 4.04% on days 15, 45, and 90, respectively. The average DO values in the CNTs group were 39.73, 45.93, and 38.05 μmol L^−1^ on days 15, 45, and 90, respectively, while the control group showed averages of 64.58, 69.40, and 56.96 μmol L^−1^. Relative to the control group, the CNTs treatment resulted in DO decreases of 50.19, 34.17, and 39.36 μmol L^−1^ within the 0–(−19 mm), 0–(−26 mm), and 0–(−15 mm) depth intervals on days 15, 45, and 90, respectively.

The average pH values for the CNTs group were 7.68, 7.57, and 7.23 on days 15, 45, and 90, in contrast to the control group values of 7.79, 7.79, and 7.55 for the same periods. Consequently, the CNTs treatment resulted in decreases in mean pH of 1.42%, 2.76%, and 4.29% compared with the control group on days 15, 45, and 90, respectively.

### 3.3. Variations in Soluble Cu and Pb in Sediment

**Figure 5:**
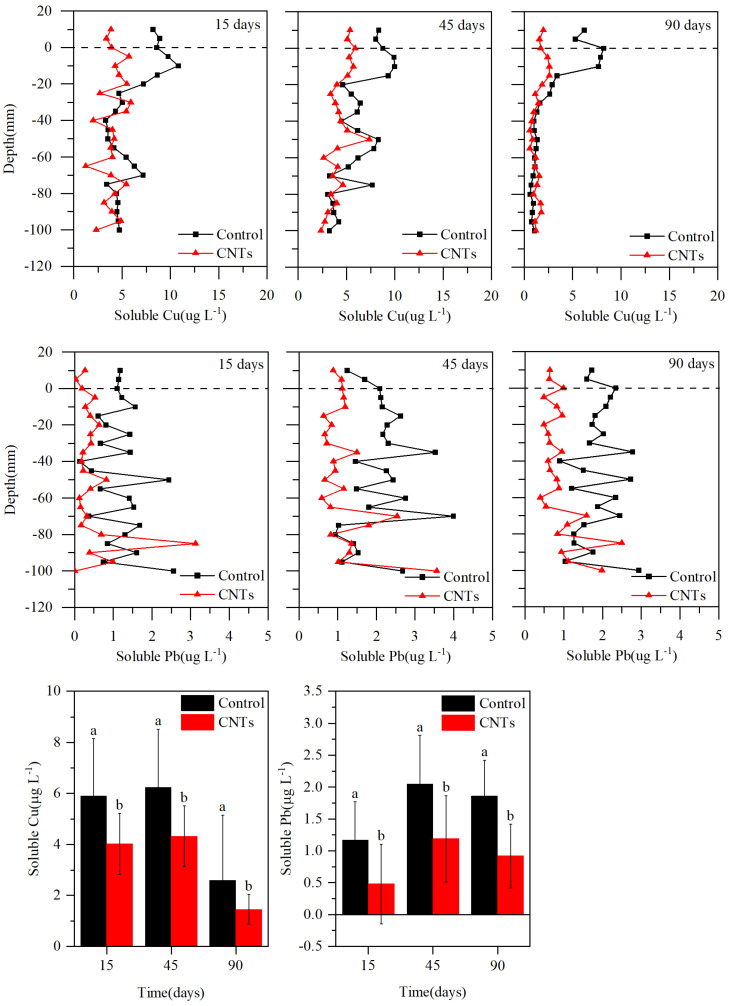
Vertical distribution of soluble Cu and Pb in sediments of the control and CNTs groups at different sampling times, the same letter between groups indicates a significant difference with the same (same letter indicates no significant difference, different letters indicate significant difference).

Figure 5 illustrates the concentrations of soluble Cu and Pb in the control and CNTs groups. On days 15, 45, and 90, the concentrations of soluble Cu in the overlying water of the control group were 8.54, 8.16, and 5.74 μg·L^−1^, respectively, whereas those in the CNTs group were 3.63, 5.24, and 1.79 μg·L^−1^. Relative to the control group, CNTs capping reduced the soluble Cu concentrations by 57.54%, 35.83%, and 68.82%, respectively. On days 15, 45, and 90, the concentrations of soluble Pb in the overlying water of the control group were 1.16, 1.47, and 0.64 μg·L^−1^, respectively, whereas those in the CNT group were 0.15, 1.00, and 0.64 μg·L^−1^. Relative to the control group, CNTs capping reduced the soluble Pb concentrations by 87.15%, 32.45%, and 61.61%, respectively. The maximum removal efficiencies of soluble Cu and Pb by CNTs capping occurred on days 15 and 90, respectively. Throughout days 15, 45, and 90, CNTs treatment significantly lowered soluble Cu and Pb concentrations relative to the control group (*p* < 0.05). Specifically, the CNTs treatment resulted in decreases of 3.80, 2.40, and 2.17 μg·L^−1^ for soluble Cu within sediment depths of 0–(−25 mm), 0–(−65 mm), and 0–(−55 mm) on days 15, 45, and 90, respectively, with the highest percentage decrease of 58.69% recorded on day 90. Similarly, CNTs capping achieved reductions in soluble Pb of 0.72, 1.33, and 1.12 μg·L^−1^ at sediment depths of 0–(−35 mm), 0–(−70 mm), and 0–(−80 mm) on days 15, 45, and 90, respectively, with the most significant reduction rate of 64.97% also occurring on day 90.

**Figure 6:**
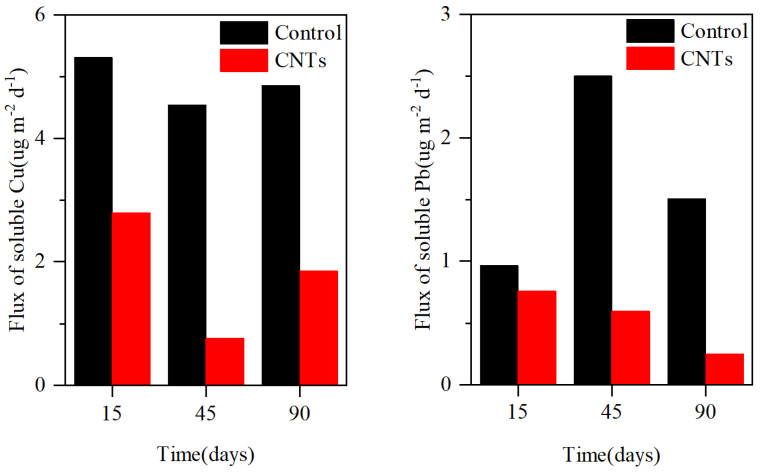
Variations in the release fluxes of soluble Cu and Pb between the control and CNTs groups at different sampling times.

Figure 6 illustrates the fluxes of soluble Cu and Pb. Over the three sampling intervals, the CNTs group consistently exhibited lower release rates compared to the control group. On days 15, 45, and 90, the control group showed soluble Cu release fluxes of 5.31, 4.55, and 4.86 μg m^−2^ d^−1^, respectively, whereas the CNTs group exhibited release fluxes of 2.80, 0.76, and 1.85 μg m^−2^ d^−1^. Specifically, on days 15, 45, and 90, the control group released 2.51, 3.78, and 3.00 μg m^−2^ d^−1^ more soluble Cu than the CNTs group. Similarly, on days 15, 45, and 90, the control group exhibited soluble Pb release fluxes of 0.97, 2.50, and 1.51 μg m^−2^ d^−1^, respectively, while the CNTs group released 0.76, 0.59, and 0.25 μg m^−2^ d^−1^ of soluble Pb. The corresponding differences in release rates between the control and CNTs groups were 0.21, 1.91, and 1.26 μg m^−2^ d^−1^ for soluble Pb on days 15, 45, and 90, respectively.

### 3.4. Speciation Changes in Cu and Pb in Sediment

**Figure 7:**
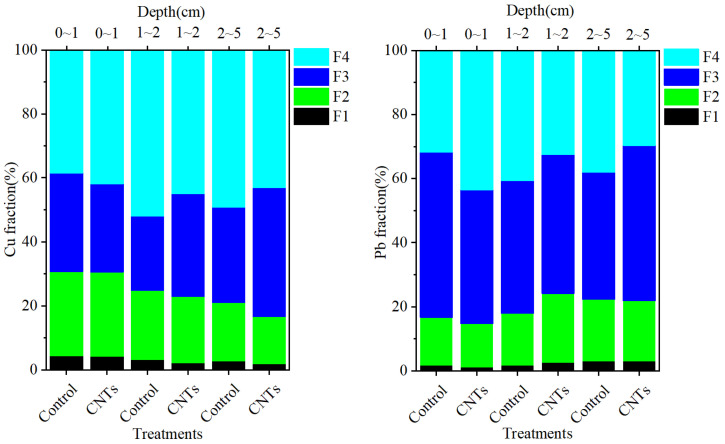
The speciation changes in Cu and Pb in sediments at different depths between the control group and the CNTs group.

Figure 7 illustrates the variations in the speciation of Cu and Pb within the sediment layers. In the control group, the mean concentrations of Cu in the F1, F2, F3, and F4 fractions within the surface 5 cm sediment layer were 3.45, 22.01, 28.06, and 46.38 mg·kg^−1^, respectively. In the CNTs group, the mean concentrations of Cu in the F1, F2, F3, and F4 fractions within the surface 5 cm sediment layer were 2.62, 19.46, 31.63, and 41.06 mg·kg^−1^, respectively. Relative to the control group, CNTs capping reduced the average Cu concentrations in the surface 5 cm sediment layer by 0.68%, 1.52%, and 3.24% for the F1, F2, and F4 fractions, respectively, while increasing the F3 fraction by 5.44%. In the control group, the mean concentrations of Pb in the F1, F2, and F4 fractions within the surface 1 cm sediment layer were 0.57, 5.18, and 34.62 mg·kg^−1^, respectively. In the surface 1 cm sediment layer of the CNTs group, the mean concentrations of Pb were 0.45, 5.37, and 39.73 mg·kg^−1^ for the F1, F2, and F4 fractions, respectively. Relative to the control group, CNTs capping reduced the F1 fraction of Pb by 0.53% and increased the F4 fraction of Pb by 11.62% in the surface 1 cm sediment layer. Additionally, the F3 fraction of Pb in the surface 5 cm sediment layer increased by 0.37% compared with the control group.

### 3.5. Mechanisms of Cu and Pb Immobilization in Sediments by CNTs

The significant reduction in soluble Cu and Pb following CNTs application indicates that this modification effectively limits the leaching of these metals from sediment (Figure 5). Nitric acid oxidation introduces numerous oxygen-rich functional groups—particularly carboxyl and lactone—onto the surface of CNTs [38,39,40,41]. This increase in oxygen-containing groups substantially enhances the adsorption capacity of multi-walled carbon nanotubes for both Cu and Pb [41,63]. Additionally, nitric acid treatment greatly improves the cation-exchange capacity of these nanotubes [41]. This is because the acidic functional groups introduced by nitric acid oxidation generate negative charges on the CNTs surface, and their oxygen atoms donate lone pairs of electrons to Cu and Pb ions, thereby enhancing the cation-exchange capacity of the nanotubes [64,65]. Therefore, proton exchange between the acidic functional groups on the CNTs surface and Cu(II) and Pb(II) in the sediment pore water is a key mechanism responsible for the decrease in dissolved copper and lead concentrations after CNTs capping [64]. The primary mechanism by which CNTs remove sediment-associated Cu and Pb involves the formation of oxide or hydroxide precipitates through chemical interactions between Cu(II) and Pb(II) and the newly introduced functional groups on the nanotube surface [41,63,65,66,67]. The Dubinin–Radushkevich (DR) model calculations corroborate this conclusion: the E values derived from the K_dr_ constant are 23.271 and 20.143 kJ mol^−1^ for Cu and Pb, respectively, both exceeding 16 kJ mol^−1^, indicating that the adsorption of Cu and Pb by CNTs is predominantly governed by chemisorption (Table 1) [58,59,60,61]. In this study, CNTs demonstrated a higher removal efficiency for soluble Pb from sediments compared to soluble Cu, as the oxidized nanotubes exhibit a stronger adsorption preference for Pb(II) over Cu(II) (Figure 5) [63]. The higher qm for Pb(II) (23.206 mg g^−1^) compared to Cu(II) (11.076 mg g^−1^) obtained from Langmuir isotherm fitting further supports this conclusion (Table 1). Likewise, the higher K_f_ value for Pb(II) (0.21) than for Cu(II) (0.201) calculated by the Freundlich model also supports this finding (Table 1).

The notable reduction in DO in sediments following the application of gauze-wrapped CNTs suggests that this capping method gradually drives the sediment toward an anoxic environment (Figure 4). Under such conditions, the reduction in Fe(III)/Mn(IV) oxyhydroxides occurs, releasing previously trapped Cu and Pb [68]. However, the Cu and Pb released during this anoxic process are rapidly adsorbed by the CNTs. The considerable decreases in the concentrations and release rates of soluble Cu and Pb in the CNTs group (Figure 5 and Figure 6) provide strong evidence for this process [69,70]. Furthermore, the mildly alkaline conditions observed in sediments after CNTs application (Figure 4) promote the uptake of both metals by the modified nanotubes [66]. Under such conditions, the CNTs surface acquires a more negative charge, thereby generating electrostatic forces that enhance their adsorption capacity for Cu and Pb [66].

**Figure 8:**
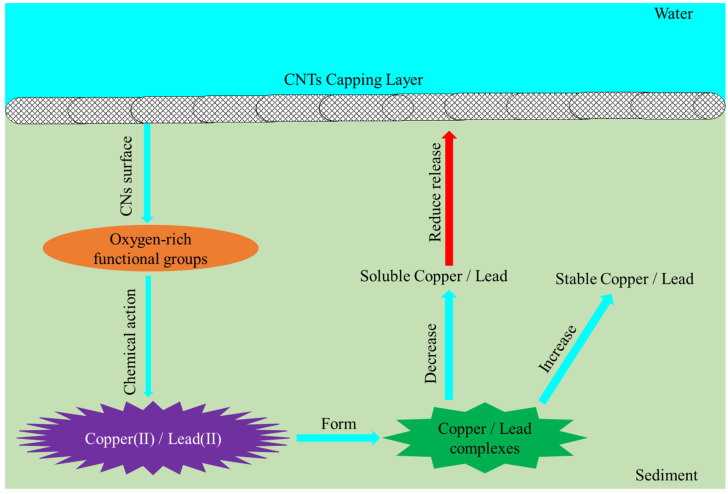
Schematic diagram of Cu(II) and Pb(II) adsorption by CNTs.

The F1 and F2 fractions of Cu and Pb are considered unstable due to their potential release via the reductive dissolution of amorphous iron oxides under reducing conditions [71]. Observations of decreased F1 and F2 levels of Cu and Pb in the surface 1 cm sediment layer following CNTs capping indicate that this treatment effectively diminishes the unstable metal fractions (Figure 7) [46]. Additionally, the increased F4 fractions of Cu and Pb in this layer demonstrate that the amendment enhances sedimentary adsorption and retention of both metals (Figure 7) [46]. CNTs capping facilitated the transformation of Cu and Pb from the F1, F2, and F3 fractions into the more stable F4 fraction within surface sediments, significantly reducing their potential release. This transformation explains the observed reductions in dissolved concentrations and release rates of Cu and Pb in the CNTs group (Figure 5, Figure 6 and Figure 8).

Previous studies on CNTs and heavy metals in sediments have primarily focused on Cd [46,47,48]. Incorporating CNTs into sediments can enhance their adsorption capacity for Cd, with optimal adsorption achieved when the CNTs-to-sediment ratio ranges from 1.25% to 10% [47]. Such incorporation can significantly reduce Cd concentrations in overlying water [48]. Owing to the strong binding affinity between CNTs and Cd, CNTs addition promotes Cd sedimentation [46]. Moreover, CNTs can reduce the F1 fraction of Cd, and higher CNTs incorporation further facilitates Cd sedimentation and immobilization [46]. However, the direct application of CNTs to sediment may pose potential risks to aquatic organisms. In contrast to these studies, we enclosed CNTs in permeable gauze, preventing direct contact between CNTs and sediment and thereby substantially reducing CNTs migration into the sediment and associated ecological risks. CNTs capping decreased soluble Cu and Pb in overlying water by up to 68.82% and 87.15%, respectively (Figure 5). Pore-water profiles showed even larger reductions in soluble Cu (58.69%) and Pb (64.97%) (Figure 5). The concurrent decline in the F1 and F2 fractions of Cu and Pb in surface sediment confirms that gauze-packed CNTs effectively immobilize these metals (Figure 7). Overall, these results highlight the efficacy of permeable-gauze-packed CNTs for in situ immobilization of Cu and Pb in contaminated sediments and provide a practical reference for their field-scale application in sediment remediation.

## 4. Conclusions

This study investigated the effects of capping sediments with CNTs on the behavior of Cu and Pb. The results showed that this method significantly reduced the concentrations of soluble Cu and Pb in the overlying water and pore water. Maximum removal efficiencies reached 68.82% for Cu and 87.15% in the overlying water, and 58.69% for Cu and 64.97% in the pore water. CNTs capping significantly reduced the release fluxes of soluble Cu and Pb. Notably, CNTs exhibited a stronger affinity for Pb than for Cu. Furthermore, CNTs capping promoted the transformation of unstable forms of Cu and Pb into more stable fractions, thereby enhancing their immobilization within sediments. In summary, CNTs represent a promising and effective strategy for mitigating the release of Cu and Pb from sedimentary environments.

## Figures and Tables

**Table 1:** Langmuir, Freundlich, and D-R model parameters for the adsorption of Cu and Pb onto CNTs.

Isotherm	Parameters	CNTs-Cu	CNTs-Pb
**Langmuir** $q_{e}=\frac{q_{m}K_{L}C_{e}}{1+K_{L}C_{e}}$	q_m_ (mg g^−1^)	11.076	23.206
	k_f_	0.004	0.003
	R^2^	0.958	0.991
**Freundlich** $q_{e}=K_{f}C_{e}^{1/n}$	K_f_	0.201	0.210
	n	1.695	1.473
	R^2^	0.921	0.974
**D-R model** $q_{e}=q_{dr}\exp(-K_{\mathrm{dr}}{\left(RT\ln(1+\frac{1}{C_{e}})\right)}^{2})$	q_m_ (mg g^−1^)	6.742	11.614
	β (mol^2^ kJ^−2^)	0.002	0.003
	E (kJ mol^−1^)	23.271	20.143
	R^2^	0.932	0.921

Where C_e_ (mg L^−1^) is the equilibrium concentration of Cu and Pb; q_e_ (mg g^−1^) represents the equilibrium adsorption amount of Cu and Pb on CNTs; K_L_ (L mg^−1^), K_f_ and 1/n are the constant of Langmuir and Freundlich, respectively; q_m_ (mg g^−1^) denotes the largest monolayer adsorption capacity; q_dr_ (mg g^−1^) is the theoretical maximum adsorption amount of Cu and Pb on CNTs; K_dr_ (mol^2^ kJ^−2^) denotes the DR constant linked to the free energy of adsorption; R (8.314 J (mol K)^−1^) represents the constant of ideal gas; T (K) is the absolute temperature; E (kJ mol^−1^) stand for the average free energy of adsorption.

## Data Availability

The raw data supporting the conclusions of this article will be made available by the authors on request.
